# Physician’s Pocket Record

**Published:** 1866-12

**Authors:** 


### Physician's Pocket Record.

Published from the office of the *Medical and Surgical Reporter*, a new aud improved "Physician's Pocket Record," comprising the following features:—A perpetual calendar; a list of new remedies, their application, doses, etc.; poisons, and their antidotes; treatment of persons asphyxiated; medicinal weights and measures; table for calculating the period of utero-gestation; table for calculating the probable duration of life; classified list of the chief articles of the materia medica, their doses, and market value; table of signs; a visiting list, day-book of accounts and daily memoranda, combined.

An Appendix, containing:—An index of patients; obstetric, vaccine, and other engagements, etc.; fee table, city and country; list of medical periodicals, with their subscription price.

It will be seen that this Pocket Record comprises some new features that cannot fail to make it the most useful and popular work of the kind in use. It is neatly, compactly, and substantially got up, of a size more suitable to the side pocket than any now before the profession.

This Pocket Record is intended to meet the wants of both city and country practitioners, and to be an indispensable companion. It is not bulky, although it contains so much material. Price, $1.50.

Address, office of the "Medical and Surgical Reporter," Philadelphia, Pa.

